# Has the IPCC’s revised vulnerability concept been well adopted?

**DOI:** 10.1007/s13280-022-01806-z

**Published:** 2022-11-21

**Authors:** Ronald C. Estoque, Asif Ishtiaque, Jagadish Parajuli, Darshana Athukorala, Yasin Wahid Rabby, Makoto Ooba

**Affiliations:** 1 Center for Biodiversity and Climate Change, Forestry and Forest Products Research Institute, Tsukuba, Japan; 2 Department of Geography, Geology and Planning, Missouri State University, Springfield, USA; 3 School of Sustainability, Arizona State University, Tempe, USA; 4 Graduate School of Life and Environmental Sciences, University of Tsukuba, Tsukuba, Japan; 5 Department of Engineering, Wake Forest University, Winston-Salem, USA; 6 Center for Climate Change Adaptation, National Institute for Environmental Studies, Tsukuba, Japan

**Keywords:** Adaptation, Climate change, Exposure, Hazard, IPCC, Risk

## Abstract

In the Third and Fourth Assessment Reports (TAR and AR4, respectively) by the Intergovernmental Panel on Climate Change (IPCC), vulnerability is conceived as a function of exposure, sensitivity, and adaptive capacity. However, in its Special Report on Managing the Risks of Extreme Events and Disasters to Advance Climate Change Adaptation (SREX) and Fifth Assessment Report (AR5), the IPCC redefined and separated exposure, and it reconceptualized vulnerability to be a function of sensitivity and capacity to cope and adapt. In this review, we found that the IPCC’s revised vulnerability concept has not been well adopted and that researchers’ preference, possible misinterpretation, possible confusion, and possible unawareness are among the possible technical and practical reasons. Among the issues that need further clarification from the IPCC is whether or not such a reconceptualization of vulnerability in the SREX/AR5 necessarily implies nullification of the TAR/AR4 vulnerability concept as far as the IPCC is concerned.

## Introduction

Adaptation to climate change and variability is one of today’s most pressing global societal challenges. In the cyclical planning process of adapting or adjusting to the actual or expected climate and its effects, climate-related vulnerability and risk assessments are an important phase because they are designed to help in the identification of adaptation options and measures (UNFCCC 2012; EC 2013; Estoque et al. in press).

**Fig. 1:**
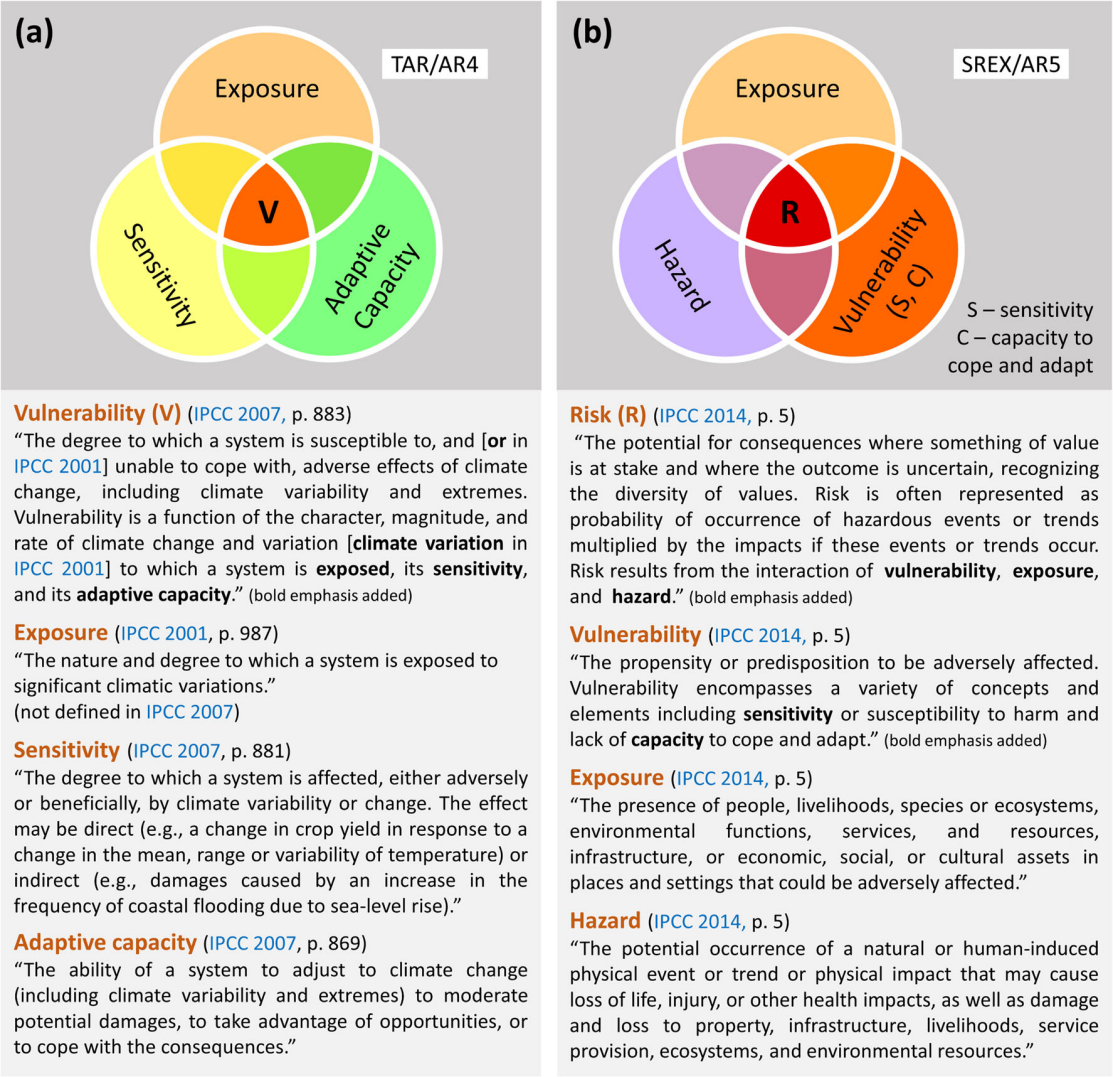
The IPCC’s climate-related impact assessment frameworks. **a** The vulnerability (V) assessment framework in the TAR/AR4, and **b** the risk (R) assessment framework in the SREX/AR5. The diagrams were drawn by the authors based on the definition of vulnerability in the TAR (IPCC 2001) and AR4 (IPCC 2007), the definition of risk and Figure SPM.1 in the AR5 (IPCC 2014), Figure SPM.1 in the SREX (IPCC 2012), and Box 1 in Foden et al. (2019)

This review focuses on vulnerability assessment. The vulnerability framework proposed by the Intergovernmental Panel on Climate Change (IPCC) in its Third (IPCC 2001) and Fourth (IPCC 2007) Assessment Reports (TAR and AR4, respectively) is widely used in climate-related vulnerability assessments (Nguyen et al. 2016, 2017; Crane et al. 2017; Aslam et al. 2018; Filho et al. 2018; Foden et al. 2019). In this framework, vulnerability is conceived as a function of exposure, sensitivity, and adaptive capacity (Fig. 1a).

However, in its Special Report on Managing the Risks of Extreme Events and Disasters to Advance Climate Change Adaptation (SREX) (IPCC 2012) and Fifth Assessment Report (AR5) (IPCC 2014), the IPCC shifted its focus to a risk-centered assessment framework, in which risk is expressed as a function of hazard, exposure, and vulnerability (Fig. 1b). As a result, exposure and vulnerability have been reconceptualized.

In the TAR/AR4, exposure is a hazard-centered concept (IPCC 2001) (indicators include heatwave duration index, drought intensity, and occurrence of floods) (Oh et al. 2017; Ducusin et al. 2019; Huynh et al. 2020; Mafi-Gholami et al. 2020), but in the SREX/AR5, it refers to exposed elements (e.g., people, assets, or ecosystems at risk) (IPCC 2012, 2014). Vulnerability, on the other hand, has become a function of sensitivity or susceptibility to harm and capacity to cope and adapt (IPCC 2014; GIZ and EURAC 2017).

The IPCC’s transition from a vulnerability to a risk framework offers new perspectives on the assessment of climate change impacts and adaptation pathways. For example, by focusing on risk, the IPCC (a) recognizes that a significant proportion of interrelated impacts are triggered by hazardous events, and thus these impacts should be appropriately addressed by the risk concept, and (b) encourages more investigative studies in risk management to determine the potential consequences of hazardous events (GIZ and EURAC 2017). The SREX/AR5 risk framework also highlights the importance of exposure and vulnerability, and it contributes to the integration of the two research realms, namely climate change adaptation and disaster risk reduction and management (GIZ and EURAC 2017; Jurgilevich et al. 2017; Estoque et al. 2020).

In this review, we attempted to measure the extent to which the IPCC’s revised vulnerability concept has been used in recent vulnerability studies to gain an understanding of whether vulnerability research synchronously responded to the conceptual advancement of vulnerability. To do this, we conducted a systematic review of climate-related vulnerability studies published within the past 4 years (January 2017–December 2020).

The literature on climate-related vulnerability is rich and continuously growing. Other reviews are available, covering a wide range of topics, from the conceptual origin and models of vulnerability (Timmerman 1981; Füssel and Klein 2006; Füssel 2007; Fellmann 2012; Giupponi and Biscaro 2015), to the relationships and integration of vulnerability with resilience (Adger 2006; Gallopín 2006), adaptation (Adger 2006; Gallopín 2006), and risk (Jurgilevich et al. 2017; Sharma and Ravindranath 2019). Some reviews have focused on indicators of vulnerability and their role in the science-policy interface (Hinkel 2011; Tonmoy et al. 2014; Nguyen et al. 2016), as well as on the sectoral and geographical applications of vulnerability assessments [e.g., social (Cutter 2003; Nguyen et al. 2017), livelihood (Hahn et al. 2009), urban (Filho et al. 2018) and coastal (Nguyen et al. 2016) regions, groundwater (Aslam et al. 2018), biodiversity (Foden et al. 2019; Pacifici et al. 2015), agriculture (Crane et al. 2017; Fellmann 2012), and forestry (FAO 2018)].

This review aims to complement these existing reviews on climate-related vulnerability by focusing on two specific questions. First, to what extent has the SREX/AR5 vulnerability concept been adopted in climate-related vulnerability assessments? Second, what factors have influenced the adoption or non-adoption of the SREX/AR5 vulnerability concept?

## Materials and methods

### Review database

We used the Web of Science (WoS) Core Collection as the source database for the review. WoS is a large database of articles, including those in the social and environmental sciences. Other databases are also available, such as Scopus (Jurgilevich et al. 2017; Tonmoy et al. 2014) and Google Scholar (de Sherbinin et al. 2019), but previous reviews have demonstrated that WoS alone can be used as a source for major systematic reviews (Runting et al. 2017; Estoque et al. 2019; Newell et al. 2019). Furthermore, the resulting total number of articles from the search process was large enough for the purpose of our review.

### Review protocol

**Fig. 2:**
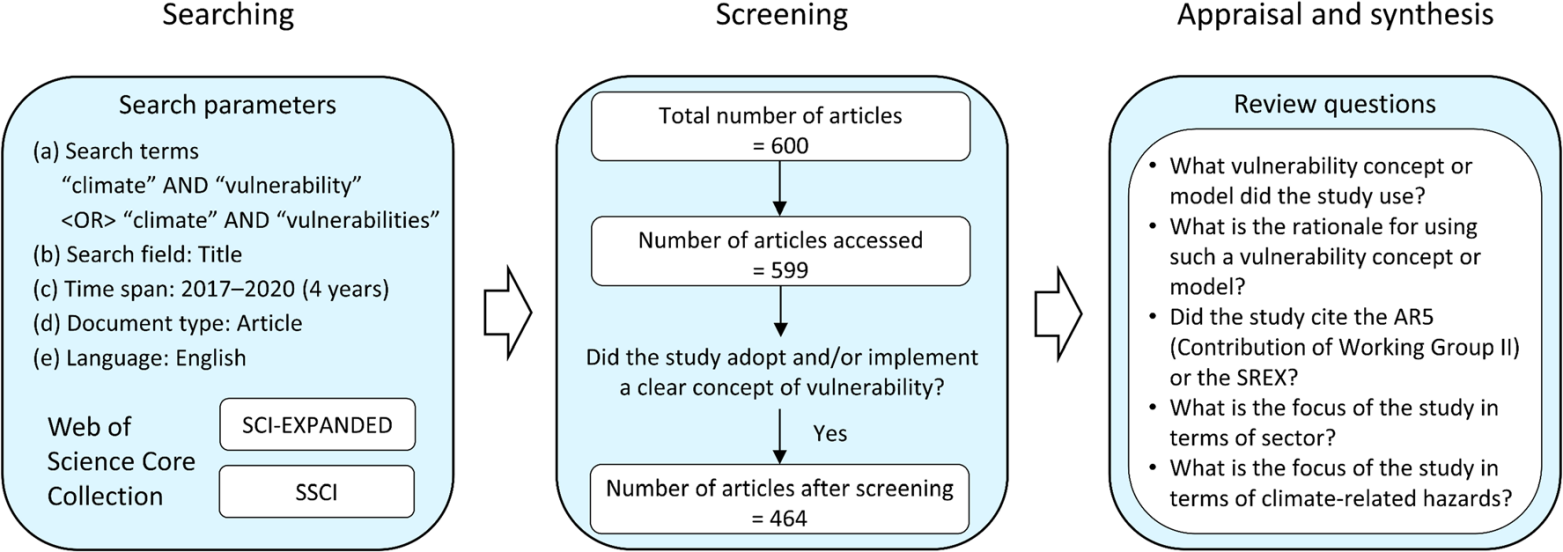
Flow of the systematic review on climate-related vulnerability assessments (January 2017–December 2020)

We performed a systematic review (Grant and Booth 2009), informed by the RepOrting standards for Systematic Evidence Synthesis (ROSES) protocol (Haddaway et al. 2018). The review process included three main steps: searching, screening, and appraisal and synthesis (Haddaway et al. 2018; Estoque et al. 2019) (Fig. 2).

#### Searching

We used two sub-databases (SCI-EXPANDED and SSCI) within the WoS Core Collection. Under “Title”, we searched for the following terms: [“climate” AND “vulnerability”] OR [“climate” AND “vulnerabilities”] (Fig. 2). We focused on “Articles” written in “English” and published within the past 4 years (1 January 2017–31 December 2020). The search resulted in 600 articles.

The commencement date (1 January 2017) was decided after taking into consideration the publication time of the SREX (2012) and the AR5 (2014). Papers published in 2015–2016 might have been based on research projects conceptualized before the publication of the AR5. Hence, the lag period was intended to allow for the dissemination of the SREX and AR5, as well as for authors to gain awareness of the latest developments in the field of climate-related vulnerability assessment, at least as far as the IPCC was concerned.

#### Screening

We were able to access all the articles except one. We reviewed each article and examined whether the article under consideration adopted and/or demonstrated a clear concept of vulnerability (Fig. 2). Many of the articles reviewed did not present a clear concept of vulnerability; for example, sometimes the word “vulnerability” was mentioned only in the title. These articles were screened out, leaving 464 articles for the next stage of the review.

#### Appraisal and synthesis

At the appraisal and synthesis stage, we answered five questions (Fig. 2; Table 1). We paid particular attention to the rationale for the choice of vulnerability concept or model adopted or used in each study. We synthesized the information obtained from this process and used it as the basis of our discussion on the possible reasons and contributing factors for the adoption or non-adoption of the SREX/AR5 vulnerability concept.

**Table 1:** Review questions and their explanations

Choices	Notes
1. What vulnerability concept or model did the study use?	
TAR/AR4, SREX/AR5, AR5-like, and other	In the TAR/AR4, vulnerability is a function of exposure, sensitivity, and adaptive capacity, in which exposure is a hazard-centered concept. In the SREX/AR5, vulnerability is a function of sensitivity and capacity to cope and adapt. The SREX/AR5-like category included studies that defined vulnerability as a function of sensitivity and capacity to cope and adapt, but without any reference to the AR5 or the SREX. Other included vulnerability concepts or models other than those mentioned above
2. What is the rationale for using such a vulnerability concept or model?	
Any reasons or explanations by the authors in this regard were considered	
3. Did the study cite the AR5 (Contribution of Working Group II) or the SREX?	
Choices	Notes
Yes or No	“Yes” means the study cited the following: Contribution of Working Group II to the Fifth Assessment Report of the Intergovernmental Panel on Climate Change (AR5), or Climate Change 2014: Impacts, Adaptation, and Vulnerability, or Managing the Risks of Extreme Events and Disasters to Advance Climate Change Adaptation (SREX), be it in the form of a synthesis report, summary for policymakers, glossary, etc. Articles citing studies that made reference to any of these sources were also included
4. What is the focus of the study in terms of sector?	
Choices	Notes
Agriculture, fisheries, forestry, biodiversity, health, energy, water, multi-sector, and other	Agriculture also included animal husbandry but excluded fisheries. Forestry also included mangrove ecosystems and urban forestry. Biodiversity also included studies focusing on (plant/animal) species and habitat vulnerability. Water also included glaciers. Multi-sector means that the study considered more than one sector. Some studies did not specify a sector; instead, they determined vulnerability in a geographic or administrative region (e.g., a coastal region, a basin, or a city or urban area). These studies were also classified under the multi-sector category. The other category included sectors other than those mentioned above
5. What is the focus of the study in terms of climate-related hazards?	
Choices	Notes
Flooding, extreme heat, drought, landslide, sea level rise, multi-hazard, and other	Flooding also included soil erosion. Extreme heat included heatwave and warming. Drought also included indicators referring to dry periods. Multi-hazard means that the study considered more than one climate-related hazard. Some studies considered changes in the intensity and pattern of more than one essential climate variable, such as temperature and rainfall, while some studies did not specify any variable but considered climate change in general. These studies were also classified under the multi-hazard category. The other category included climate-related hazards other than those explicitly mentioned above, including wildfires, pests, and windstorms

## Results and discussion

### Recent trend in climate-related vulnerability assessment

**Fig. 3:**
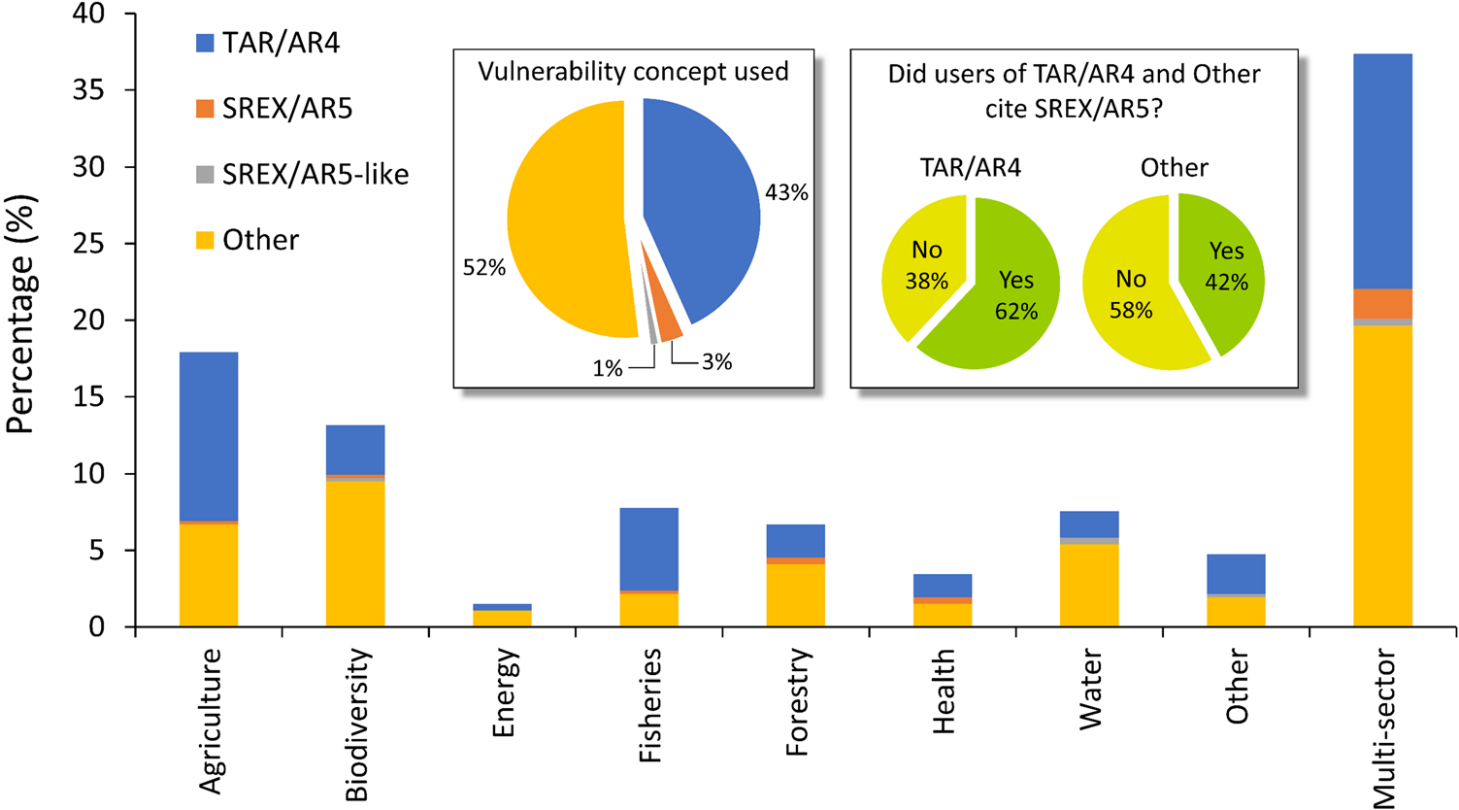
Climate-related vulnerability assessments (January 2017–December 2020). The stacked column graph shows the distribution of vulnerability studies (*n* = 464) across sectors subdivided according to the vulnerability concept used. The inset pie chart on the left summarizes the proportion of studies that adopted and/or implemented the vulnerability concepts. The inset pie charts on the right show the proportion of studies that adopted and/or implemented the TAR/AR4 and other vulnerability concepts and that cited or did not cite the SREX/AR5 vulnerability concept. See Table 1 for details

The SREX/AR5 vulnerability concept was used in the IPCC’s 1.5 °C Special Report (IPCC 2018) and was regarded as influential (Barnett 2020). Yet, our results indicate that this revised vulnerability concept has not been well adopted in climate-related vulnerability studies across sectors worldwide and that its influence in the field of climate-related assessment has so far been minimal. Of the 464 research articles that we reviewed, 201 (43%) employed the TAR/AR4, 241 (52%) used other vulnerability concepts, and only 16 (3%) adopted and/or implemented the SREX/AR5 vulnerability concept (Fig. 3). In general, our findings are consistent with earlier observations. For example, some studies have noted that the IPCC’s revised vulnerability concept has received little attention (Borges et al. 2019; Foden et al. 2019) and that the TAR/AR4 vulnerability concept continues to predominate (Pinnegar et al. 2019; Timberlake and Schultz 2019) and to be used across vulnerability studies (Nguyen et al. 2016; Crane et al. 2017; Filho et al. 2018; Aslam et al. 2018).

### Reasons for low adoption of the IPCC’s revised vulnerability concept

Most of the studies that we reviewed did not explain the rationale for their adoption and/or implementation of a particular vulnerability concept or model. Because of this, we could not synthesize in this review the plausible *theoretical* reasons behind the low adoption of the SREX/AR5 vulnerability concept. Such reasons may include any observed advantages/strengths and disadvantages/weaknesses of the SREX/AR5 vulnerability framework for a particular vulnerability assessment. Nonetheless, based on our synthesis, we have identified a number of possible *technical and practical* reasons for the low adoption of the IPCC’s revised vulnerability concept, including researchers’ preference, possible misinterpretation, possible confusion, and possible unawareness. We believe these technical and practical reasons are as important as any plausible theoretical reasons. For instance, if the researchers were not aware of the existence of the IPCC’s revised vulnerability concept, then there would be no discussion about the theorical reasons for its low adoption.

In the following discussion, “[*n*]” refers to the article code assigned to the study and referred to in Tables 2, 3 and 4.

**Table 2:** List of reviewed articles that are referred to in the discussion by article code (i.e., [*n*]). Studies listed in Tables 3 and 4 are not shown here

Article code	Focus climate-related hazard	Focus sector	Vulnerability model	Did it cite the SREX/AR5?	References
2	Multi-hazard	Agriculture	TAR/AR4	Yes	Xu et al. (2020)
33	Multi-hazard	Agriculture	TAR/AR4	Yes	Dhamija et al. (2020)
40	Multi-hazard	Multi-sector	Other	Yes	Johns et al. (2020)
60	Multi-hazard	Agriculture	TAR/AR4	Yes	Schneiderbauer et al. (2020)
89	Sea level rise	Forestry	TAR/AR4	Yes	Cinco-Castro and Herrera-Silveira (2020)
113	Sea level rise	Other	TAR/AR4	Yes	Shi and Varuzzo 2020)
144	Multi-hazard	Multi-sector	Other	No	Zadkovic et al. (2021)
146	Multi-hazard	Multi-sector	TAR/AR4	No	Schilling et al. (2020)
152	Multi-hazard	Multi-sector	TAR/AR4	Yes	Zhang et al. (2020)
161	Multi-hazard	Agriculture	TAR/AR4	Yes	Adzawla and Baumüller (2021)
189	Multi-hazard	Multi-sector	TAR/AR4	Yes	Gerlak and Greene (2019)
194	Extreme heat	Fisheries	Other	Yes	Troia and Giam (2019)
195	Multi-hazard	Multi-sector	TAR/AR4	Yes	Gupta et al. (2019)
201	Multi-hazard	Forestry	Other	No	Wang et al. (2019)
207	Multi-hazard	Other	TAR/AR4	Yes	McIntosh and Becker (2019)
213	Multi-hazard	Multi-sector	Other	Yes	Owusu and Nursey-Bray (2019)
214	Multi-hazard	Fisheries	TAR/AR4	Yes	Greenan et al. (2019)
218	Multi-hazard	Biodiversity	Other	No	Rinnan and Lawler (2019)
224	Multi-hazard	Agriculture	TAR/AR4	Yes	Lokonon (2019)
228	Multi-hazard	Biodiversity	TAR/AR4	Yes	Borges et al. (2019)
233	Drought	Water	Other	No	Kim et al. (2019)
234	Multi-hazard	Fisheries	TAR/AR4	Yes	Crozier et al. (2019)
237	Multi-hazard	Agriculture	TAR/AR4	Yes	Huong et al. (2019)
239	Multi-hazard	Multi-sector	TAR/AR4	Yes	Zhang et al. (2019)
242	Multi-hazard	Multi-sector	Other	No	Formetta and Feyen (2019)
261	Multi-hazard	Health	TAR/AR4	Yes	Bae et al. (2019)
273	Multi-hazard	Multi-sector	TAR/AR4	Yes	He et al. (2019)
296	Flooding	Multi-sector	TAR/AR4	Yes	Dogra et al. (2019)
299	Multi-hazard	Multi-sector	Other	Yes	Apreda et al. (2019)
319	Multi-hazard	Biodiversity	TAR/AR4	Yes	Foden et al. (2019)
381	Flooding	Forestry	TAR/AR4	Yes	Sam and Chakma (2018)
421	Multi-hazard	Forestry	TAR/AR4	Yes	Menezes et al. (2018)
453	Multi-hazard	Other	TAR/AR4	Yes	Jedd et al. (2018)
460	Multi-hazard	Agriculture	TAR/AR4	Yes	Steiner et al. (2018)
503	Multi-hazard	Multi-sector	TAR/AR4	No	Nguyen et al. (2017)
517	Multi-hazard	Fisheries	TAR/AR4	Yes	Monnereau et al. (2017)
553	Multi-hazard	Multi-sector	TAR/AR4	Yes	Shukla et al. (2017)
560	Multi-hazard	Biodiversity	TAR/AR4	Yes	Culp et al. (2017)
598	Multi-hazard	Agriculture	TAR/AR4	Yes	Wiréhn et al. (2017)

**Table 3:** List of reviewed articles that adopted and/or implemented the SREX/AR5 vulnerability concept

Article code	Focus climate-related hazard	Focus sector	Vulnerability model (Operationalization of the IPCC’s revised vulnerability concept)	Remarks	References
10	Extreme heat	Health	f(sensitivity, adaptive capacity)	In the context of risk. Used the SREX, together with another reference	Jagarnath et al. (2020)
21	Multi-hazard	Forestry	f(susceptibility, lack of adaptive capacity)	In the context of risk. Used the IPCC’s 1.5 °C special report. That report is based on the SREX/AR5	Lecina-Diaz et al. (2021)
62	Flooding	Multi-sector	f(susceptibility, lack of resilience), where the latter is: f(lacking capacity to anticipate, cope, and recover)	In the context of risk. Used the AR5, together with another framework	Leis and Kienberger (2020)
91	Multi-hazard	Multi-sector	f(social, economic, environmental indicators)	Used the SREX. Indicators were not categorized into sensitivity and capacity to cope and adapt	Orozco et al. (2020)
169	Multi-hazard	Fisheries	f(sensitivity and adaptability)	Based on a previous study	Chen et al. (2020)
271	Drought	Multi-sector	f(28 factors from six different sectors: land use, economy, health, energy and infrastructure, social, and water resources)	In the context of risk. Used the SREX. Factors were not categorized into sensitivity and capacity to cope and adapt	Ahmadalipour et al. (2019)
278	Multi-hazard	Multi-sector	f(community-scale socioeconomic or demographic indicators)	In the context of risk. Used the SREX. Indicators were not categorized into sensitivity and capacity to cope and adapt	Spangler et al. (2019)
363	Multi-hazard	Multi-sector	f(sensitivity, adaptability)	Used the AR5	Gao et al. (2018)
388	Multi-hazard	Multi-sector	Vulnerable households are those that fall below a pre-set poverty line with a certain probability	Used the AR5. Indicators were not categorized into sensitivity and capacity to cope and adapt	Angelsen and Dokken (2018)
423	Multi-hazard	Biodiversity	f(sensitivity, adaptive capacity)	In the context of risk. Used the AR5	Jones and Cheung (2018)
457	Multi-hazard	Forestry	f(sensitivity, adaptive capacity)	Used the AR5	Halofsky et al. (2018)
476	Multi-hazard	Multi-sector	f(social, economic, environmental indicators)	Used the SREX. Indicators were not categorized into sensitivity and capacity to cope and adapt	Duvat et al. (2017)
518	Multi-hazard	Multi-sector	f(sensitivity, adaptive capacity)	Used the AR5	Tapia et al. (2017)
530	Multi-hazard	Health	f(socio-economic indicators)	Used the AR5. Indicators were not categorized into sensitivity and capacity to cope and adapt	Navi et al. (2017)
540	Multi-hazard	Agriculture	f(susceptibility, capacity)	Used the AR5	Jones et al. (2017)
573	Other	Multi-sector	f(the degree to which household income is affected by variation in rainfall)	Used the AR5. The study focused on economic vulnerability	Flatø et al. (2017)

**Table 4:** List of reviewed articles that adopted and/or implemented a SREX/AR5-like vulnerability concept

Article code	Focus climate-related hazard	Focus sector	Vulnerability model	Remarks	References
41	Multi-hazard	Biodiversity	f(climate sensitivity, adaptive capacity)	Referred climate sensitivity to a study published in 2003 and adaptive capacity to a study published in 2019	Valencia et al. (2020)
149	Multi-hazard	Multi-sector	f(susceptibility, resilience)	Based on the authors’ review of the literature	Jhan et al. (2020)
270	Multi-hazard	Multi-sector	f(sensitivity, adaptation)	Proposed by the authors, arguing that “exposure indexes are hard to consider at the national scale, not only because the contribution of temperatures and precipitation varies among countries but also because it is hard to judge the negative or positive impact of exposure [citing one study]” (p. 217)	Li et al. (2019)
316	Multi-hazard	Other	f(sensitivity, adaptive capacity)	Based on a study published in 2011	Cowood et al. (2019)
487	Multi-hazard	Water	f(sensitivity, adaptability)	Based on a study published in 2012	(Xia et al. (2017)
586	Multi-hazard	Water	f(sensitivity, adaptability)	Based on a study published in 2012	Shi et al. (2017)

### Researchers’ preference

The conceptual framing of vulnerability varies across fields of study, and scholars tend to prefer a framework that is already relatively more established in their respective fields. For example, in a separate review on species vulnerability, the authors eschewed the SREX/AR5 in favor of the TAR/AR4 vulnerability concept because the TAR/AR4 vulnerability concept had been widely adopted by the conservation community, with little attention paid to the IPCC’s revised vulnerability concept [319]. This observation was also echoed by other scholars [228]. Other researchers selected the TAR/AR4 vulnerability concept because they wanted to compare their studies with other previous studies [146, 517].

Furthermore, many of the studies that we reviewed anchored their vulnerability assessments on the social vulnerability index [503], livelihood vulnerability index [237], and integrated [218] and trait-based [228] frameworks for assesing species vulnerability, all of which are based on the TAR/AR4 conceptual framing of vulnerability (Williams et al. 2008; Hahn et al. 2009; Foden et al. 2013; Foden et al. 2013; Nguyen et al. 2017). Other scholars used the TAR/AR4 vulnerability concept because their research projects were conceptualized before the publication of the AR5 [60, 517]. There were also studies that implemented vulnerability frameworks other than those of the TAR/AR4 and SREX/AR5 [e.g., 144, 201, 233, 242]. With regard to social vulnerability, for example, some researchers argue that the IPCC’s vulnerability concept in general has significant limitations because it “downplays the degree to which different social groupings experience hazards or risks”, and that a contextual vulnerability from a political ecology perspective is more appropriate [213].

### Possible misinterpretation

Many researchers are aware of the SREX/AR5 as indicated by their citations (Fig. 3) and discussion of the reports, but some of them have operationalized the revised vulnerability concept according to their own interpretations. For example, in a study of vulnerability and the impacts of heatwaves and flooding on urban systems, the SREX/AR5 vulnerability concept was operationalized by considering overall vulnerability as a function of intrinsic vulnerability and exposure [299]. Some researchers, after acknowledging that the IPCC had revised its concept of vulnerability in the SREX/AR5, argued that the three components of vulnerability in the TAR/AR4 remain relevant and can still be used [e.g., 207, 228, 598]. Other researchers have claimed that the AR5 vulnerability concept originates from the AR4 vulnerability concept [33] and that it remains as a function of exposure, sensitity, and adaptive capacity [33, 224].

### Possible confusion

Among the studies that cited the SREX/AR5 (Fig. 3) but implemented another vulnerability model or framework, we observed some indications of possible confusion. For example, many related studies (e.g., in the contexts of the global framework for climate services [189], agriculture [2], agro-ecological zones [553], forestry [381], coastal regions [152], livelihood [161], health [296], fiscal planning [113], urbanization [273], tourism [453], mangrove ecosystems [89], fisheries [214], and migratory birds [560]) defined vulnerability as a function of exposure, sensitivity, and adaptive capacity, but the definition explicitly referred to the SREX/AR5 (especially AR5). In a study on forest landscape vulnerability to climate change, researchers also claimed that the AR5 “[divided] vulnerability to climate stressor into three domains”, referring to the same vulnerability components in the TAR/AR4 [381]. Other studies anchored their vulnerability concept to the SREX/AR5 but ultimately defined it as a function of these same three components [e.g., 195, 224, 421, 460].

### Possible unawareness

A large proportion of the studies we reviewed did not cite or even mention the SREX/AR5 (Fig. 3). Although it might not always be the case, such non-citation is a possible indication of unawareness among climate-related vulnerability researchers of the IPCC’s revised vulnerability concept. That said, citation of the SREX/AR5 does not necessarily mean the authors were aware of the revised vulnerability concept. For example, many of the studies that cited the SREX/AR5 did not cite the reports for its vulnerability concept, but rather cited them for other issues, such as the impacts of climate change in general [e.g., 40, 194, 234, 239, 261]. The authors of the studies cited above (under “Possible confusion”) who categorically referred to the TAR/AR4’s three original vulnerability components as part of the SREX/AR5 might also have been unaware of the reconceptualization of the IPCC’s vulnerability concept.

The reconceptualization of the vulnerability concept by the IPCC was not well discussed in the SREX/AR5, and this might have contributed to its low adoption rate. In addition, now that vulnerability has been reconceptualized, it is unclear what will happen to the TAR/AR4 vulnerability concept/framework. For example, is the SREX/AR5 vulnerability concept intended for risk assessment, whereas the TAR/AR4 vulnerability concept can still be used for a stand-alone vulnerability assessment? (We discuss this issue in the next section.) These basic questions need some clarification. It would have been better and clearer had the operationalization and implications of the IPCC’s revised vulnerability concept been well discussed in the SREX/AR5. Of the studies that did adopt and/or implement the IPCC’s revised vulnerability concept, many did so in the context of risk (Table 3). This is not surprising because the IPCC’s reconceptualization of vulnerability happened with the IPCC’s adoption of a risk framework. Some of these studies framed their vulnerability assessment based on the SREX/AR5 [e.g., 271, 518], while some studies were complemented by other frameworks or models [e.g., 10, 62].

### A call for further clarification

We recognize that vulnerability is an important subject across many fields of study, including but not limited to political ecology, human ecology, human geography, disaster science, and climate change research, and that it is a complex, multidimensional concept that is still evolving. Climate-related vulnerability assessments may be anchored to different frameworks for a variety of reasons, ranging from the conceptual framing of the assessment to the preference of researchers. At the fundamental level, however, it is necessary to have a clear definition of vulnerability so that (1) an assessment framework can be formulated; (2) vulnerable ecosystems, assets, and populations can consequently be more accurately determined; and (3) plausible adaptation options can be properly identified.

Because IPCC reports like the TAR/AR4 and SREX/AR5 summarize and synthesize the state of knowledge about climate change and its impacts, they not only influence climate-related research worldwide, but also the formulation of international standards (e.g., ISO 1409: Adaptation to climate change—Guidelines on vulnerability, impacts and risk assessment). However, both in the SREX and AR5, the operationalization and implications of the IPCC’s revised vulnerability concept have not been explicitly explained. Considering that such a reconceptualization of vulnerability is a major conceptual advancement (GIZ and EURAC 2017; Jurgilevich et al. 2017; Sharma and Ravindranath 2019), at least a sub-section in the SREX/AR5 should have been devoted to clarifying important issues that might influence its interpretation, adoption, and operationalization.

Among the critical issues that need clarification are the following: Does the redefinition of exposure and vulnerability in the SREX/AR5 necessarily imply nullification of the TAR/AR4 vulnerability concept as far as the IPCC is concerned? Or should the two concepts of vulnerability be interpreted and used independently as our review findings seemed to indicate is being done? That is, should the TAR/AR4 vulnerability concept be used for stand-alone vulnerability assessments and the SREX/AR5 vulnerability concept for vulnerability assessments in the context of risk? Or should the two concepts or models of vulnerability be used together in an integrated manner, and if so, how? These questions should not be interpreted as asking the IPCC to be prescriptive. Rather, they should simply be considered as questions that aim to bridge the knowledge gap resulting from the reconceptualization of vulnerability by the IPCC.

In the recently released Sixth Assessment Report (AR6) by the IPCC’s Working Group II (Impacts, Adaptation and Vulnerability), the SREX/AR5 risk framework has been adopted. To address climate change risks, the report emphasizes climate resilient development pathways with a strong focus on the interactions among coupled climate systems, ecosystems (including their biodiversity), and human society (IPCC 2022). In the report, the IPCC’s revised vulnerability concept was adopted: “Vulnerability in this report is defined as the propensity or predisposition to be adversely affected and encompasses a variety of concepts and elements, including sensitivity or susceptibility to harm and lack of capacity to cope and adapt” (p. 5) (IPCC 2022). Unfortunately, the questions raised above remain unclarified. Such a clarification, if and when it is done, can help advance the science and practice of climate-related vulnerability assessment across sectors worldwide, which is needed to help address the growing challenges of climate adaptation.

### Limitations and prospects

We acknowledge that the results of this review are largely reliant on the search terms used, which are focused on climate-related vulnerability assessment. The non-inclusion of other related terms such as hazard, exposure, risk, disaster, and adaptation, among others, narrowed the scope of the review to the field of climate-related vulnerability assessment. For this specific field, the results revealed overwhelming evidence that the IPCC’s revised vulnerability concept has not been well adopted. The IPCC’s revised vulnerability concept, together with hazard and the redefined concept of exposure, are contained within the broader concept of risk as defined by the IPCC (Fig. 1b). Notably, many of the studies that employed the IPCC’s revised vulnerability concept performed vulnerability assessment in the context of risk following the IPCC’s risk framework (Table 3). This means that had we used other terms (e.g., “risk”) in the search process, other studies would have also been captured (e.g., Mysiak et al. 2018; Akter et al. 2019; Estoque et al. 2020). This points to the importance of the question raised above about whether the TAR/AR4 vulnerability concept should be used for stand-alone vulnerability assessments, and the SREX/AR5 vulnerability concept should be used for vulnerability assessments in the context of risk.

A possible follow-up to this review would include other relevant search terms, as well as an expanded time period to include more recent studies. In addition, this review focused on plausible technical and practical reasons for why the IPCC’s revised vulnerability concept has not been well adopted, but another way forward is to look into theoretical reasons. Future works in this area can build upon other related works (e.g., Jurgilevich et al. 2017; Sharma and Ravindranath 2019; Ishtiaque et al. 2022). Directly consulting with authors of vulnerability studies, as well as leading experts in the field (e.g., via a questionnaire survey) might also help shed light on the theoretical reasons for the adoption or non-adoption of the SREX/AR5 vulnerability concept in climate-related vulnerability assessments.

## Conclusions

In this review, we attempted to determine the extent to which the IPCC’s revised vulnerability concept has been used in recent vulnerability studies to understand whether vulnerability research synchronously responded to the conceptual advancement of vulnerability. We found that the IPCC’s revised vulnerability concept has not been well adopted and that its influence in the field of climate-related vulnerability assessment has so far been minimal. While we could not identify the theoretical reasons for this, we identified researchers’ preference as well as possible misinterpretation, confusion, and unawareness as potential technical and practical reasons behind this trend. The lack of a focused discussion of the operationalization and implications of the revised vulnerability concept in the SREX/AR5 might have contributed to its low level of adoption. Overall, our review findings indicated that the TAR/AR4 vulnerability concept has been adopted for stand-alone vulnerability assessments, whereas the SREX/AR5 vulnerability concept has been used for vulnerability assessments in the context of risk. We therefore pose the following question: Was having two concepts of vulnerability part of the IPCC’s rationale when it changed its impact assessment framework from one that focused on vulnerability to one that focused on risk and reconceptualized the ideas of vulnerability and exposure? There are several issues that need further clarification from the IPCC, including whether or not such a reconceptualization of vulnerability in the SREX/AR5 necessarily implies nullification of the TAR/AR4 vulnerability concept.
